# Soybean meal and poultry offal meal effects on digestibility of adult dogs diets: Systematic review

**DOI:** 10.1371/journal.pone.0249321

**Published:** 2021-05-27

**Authors:** Karoline Vanelli, Angela Cristina Fonseca de Oliveira, Cristina Santos Sotomaior, Saulo Henrique Weber, Leandro Batista Costa

**Affiliations:** Programa de Pós-graduação em Ciência Animal, Pontifícia Universidade Católica do Paraná, Curitiba, Paraná, Brasil; University of Illinois, UNITED STATES

## Abstract

Soybean meal and poultry offal meal are protein ingredients commonly used in the formulation of commercial diets for dogs. However, there remains great variability in the data on the digestibility of each protein source. This systematic review study aimed to examine the intake, apparent nutrient digestibility coefficients and fecal output of protein sources (soybean meal and poultry offal meal) in adult dog food as reported in published studies. The article search was conducted in August 2018 in the PUBMED, SciELO, Science Direct and AGRIS indexing databases. The literature search was performed using "digestibility", "source protein" and "dog" as the main key terms combined with sub-terms to broaden the scope of the search. Criteria were defined for readability, exclusion and inclusion of articles. Results were organized in groups according to the search in the indexing databases, totaling 1,414 articles. After the works were selected following the inclusion criteria, 17 articles were evaluated in this review. According to most studies, plant-based ingredients have a less variable nutritional composition than animal-derived ingredients and poultry offal meal increases the digestibility coefficients of nutrients and energy and reduces fecal dry matter production. Factors inherent to raw-material origin, ingredient and food processing, as well as the high heterogeneity of the methodologies evaluated in the studies are directly related to the obtained results. To ensure a more accurate evaluation of the quality and of effects on the digestibility of protein sources, we recommended that articles include ingredient processing data and that the variables be evaluated under standardized study conditions.

## Introduction

To determine the quality of a dog food, one must consider the nutritional requirements of these animals as well as the ingredients (protein sources, mainly), metabolizable energy content, palatability and digestibility of the product. Dogs require high levels of dietary protein, which can vary depending on the size and age of the animal. A minimum inclusion of 18% crude protein is recommended for adult and medium-sized dogs or 25% in the case of puppy, pregnant and lactating dogs [1].

A complete and adequate diet has an ideal balance of essential amino acids from a set of protein sources of plant and animal origins. Animal by-products usually provide ideal concentrations of amino acids and proteins, but the quality of these nutrients can vary widely between sources. This variability may be related to different factors, such as particle size; levels of inclusion in the food; methodology used to estimate amino acid digestibility, composition and bioavailability; and processing [2]. Animal meals (tallow, oil, fat and flour) are generated during the rendering process, which consists of the heat treatment and recovery of wastes from slaughterhouses and meat-packing plants, which in turn must be free of materials foreign to their composition and pathogenic microorganisms [3, 4]. In addition, these by-products are subjected to a second processing step (extrusion) that takes place during the production of the commercial food. The extrusion process promotes physical-chemical changes in the ingredients that enhance the nutritional value and increase the digestibility of the food [5]. However, the quality of the heat treatment and extrusion is important, because, depending on how these processes are carried out, they may directly interfere with the nutritional composition of the raw material, increasing or reducing the bioavailability of amino acids, palatability and nutrient digestibility [6].

Among animal by-products, offal meal is the ingredient most widely employed by industries in the formulation of commercial dog foods [7]. The product results from the cooking, pressing and milling of poultry offal, where the inclusion of heads and feet is allowed. It must not contain feathers, except those which can occur unintentionally. All parts resulting from slaughter can be included, but these must not contain hatchery waste or contamination with eggshells. Inclusion of these parts and other foreign materials characterizes adulteration. The composition of offal meal is extremely variable, and its protein content can vary from 55 to 65% [3]. Despite being an excellent source of essential and non-essential amino acids [7], diets based on this by-product may have their digestibility reduced, as the bioavailability of nutrients can be influenced by the level of inclusion of different animal tissues and by processing [8, 9].

Although most industries recommend the inclusion of animal-derived meals as a primary source of protein in the formulation of dog diets, several studies indicate that plant-based protein sources with an adequate essential amino acid profile can increase the nutritional value of the diet [8–10]. In general, plant-based ingredients have a less variable composition when compared with products of animal origin [9].

When included in the diet in association with animal proteins, soy protein complements the essential amino acid profile and increases the nutritional quality of the food [9]. Additionally, because it is largely available, it contributes to reducing the production costs of commercial foods [11, 12]. Soybean meal is a by-product derived from the grinding, heating and extraction of the lipid content of the grain [9]. In addition to its high energy value, it is a source of protein and essential fatty acids (linoleic acid, mainly) [10]. However, soybean meal contains several antinutritional factors such as protease inhibitors, phytates, lecithins and non-starch polysaccharides [13], which are not digested by dogs, which may limit its use in diets for this species. Extrusion inactivates the protease inhibitors present in the meal due to the high temperatures used in the process (110 to 180°C) [14]. Nevertheless, the extrusion of the feedstuff can contribute to the over-processing of the meal, denaturing its proteins and compromising its amino acid bioavailability. On the other hand, under-processing will not remove anti-nutritional factors such as oligosaccharides, which cause gastrointestinal discomfort (flatulence), contribute to the formation of low-quality stools, dilute the energy of the diet and reduce the palatability and digestibility of the food [15–17]. To date, no systematic review or meta-analysis has been carried out to investigate the nutritional composition and digestibility of protein ingredients in dogs. Therefore, this study proposes to examine and compare the effects of including soybean meal and poultry offal meal in adult dog food on the parameters of intake, apparent nutrient digestibility coefficient and fecal characteristics (fecal production and fecal dry matter) through a systematic review.

## Materials and methods

### Study protocol

This systematic review study was undertaken to evaluate publications related to nutritional and digestibility characteristics of protein sources (soybean meal and poultry offal meal) commonly used in commercial adult dog food. The study was developed by five authors and began in August 2018. The developed protocol followed the requirements established by the Preferred Reporting Items for Systematic Reviews and Meta-Analyses (PRISMA) [18]. Although the protocol was not previously registered, this work was conducted similarly to other recent articles published in a systematic review and meta-analysis format [19, 20].

### Source and research information

The studies that make up this review were found through searches in electronic databases and in articles’ reference lists. The searches were performed in the PUBMED, Scielo, Science Direct and AGRIS indexing bases. The keywords used were *(apparent total tract digestibility OR digestibility OR nutrition*) AND (*protein sources OR dietary protein OR protein ingredients OR animal protein OR vegetal protein*) AND (*dog OR canine OR adult dog*). Combinations between keywords were always made in sets of three to ensure that more studies were found. The terms and sub-terms used for the search of references are listed in Table 1. No restrictions were applied as to year of publication and language, and the last data search was performed on 11/17/2018.

**Table 1:** Keywords, terms and sub-terms.

Terms	Sub- terms
Apparent total tract digestibility	Ash, crude protein, crude energy, digestibility, fecal, intake
Dogs	Adult dogs, pet
Nutrition	Diet, canine nutrition, metabolismo
Protein sources	Protein ingredient, dietary protein, protein food, animal ingredient, vegetal sources, by-products, co-products

### Selection of studies and construction of databases

As previously mentioned, this systematic review was not limited to studies published in English and there was no restriction on the year of publication. The following materials were excluded: book references, book chapters, literature reviews, articles that were not available in full and articles whose author could not be contacted to obtain detailed data in order to preserve the reliability of the results found.

This review only included studies developed with healthy, adult (1–6 years old) and medium-sized (10–25 kg) dogs. Animal age, size and physiological status are known to determine its nutritional requirements as well as the digestibility of the food, and these differences are well known between puppies and adult dogs [21, 22].

In addition to these parameters, studies that evaluated only soybean meal and poultry offal meal in commercial dog foods were included in this review. The studies must necessarily present the parameters of intake levels and total apparent digestibility coefficient (ADC) of at least one of the following variables: dry matter (DM), organic matter (OM), crude protein (CP), ether extract (EE), gross energy (GE) and metabolizable energy (ME). Studies in which the diet was supplemented with sources of prebiotic fibers, probiotics, enzymes and any other nutrient or additive that interfered with the digestibility of the food were dismissed. When protein sources other than those of interest in this review were tested in the same experimental trial, only groups with soybean meal and poultry offal meal were considered, regardless of their inclusion levels. When the digestibility of protein sources in dogs of different ages and sizes was evaluated in the same protocol, only the groups of adult and medium-sized dogs were classified. In comparative studies between collection methods (total or ileal), only the groups evaluated by the total fecal collection method were included.

**Fig 1:**
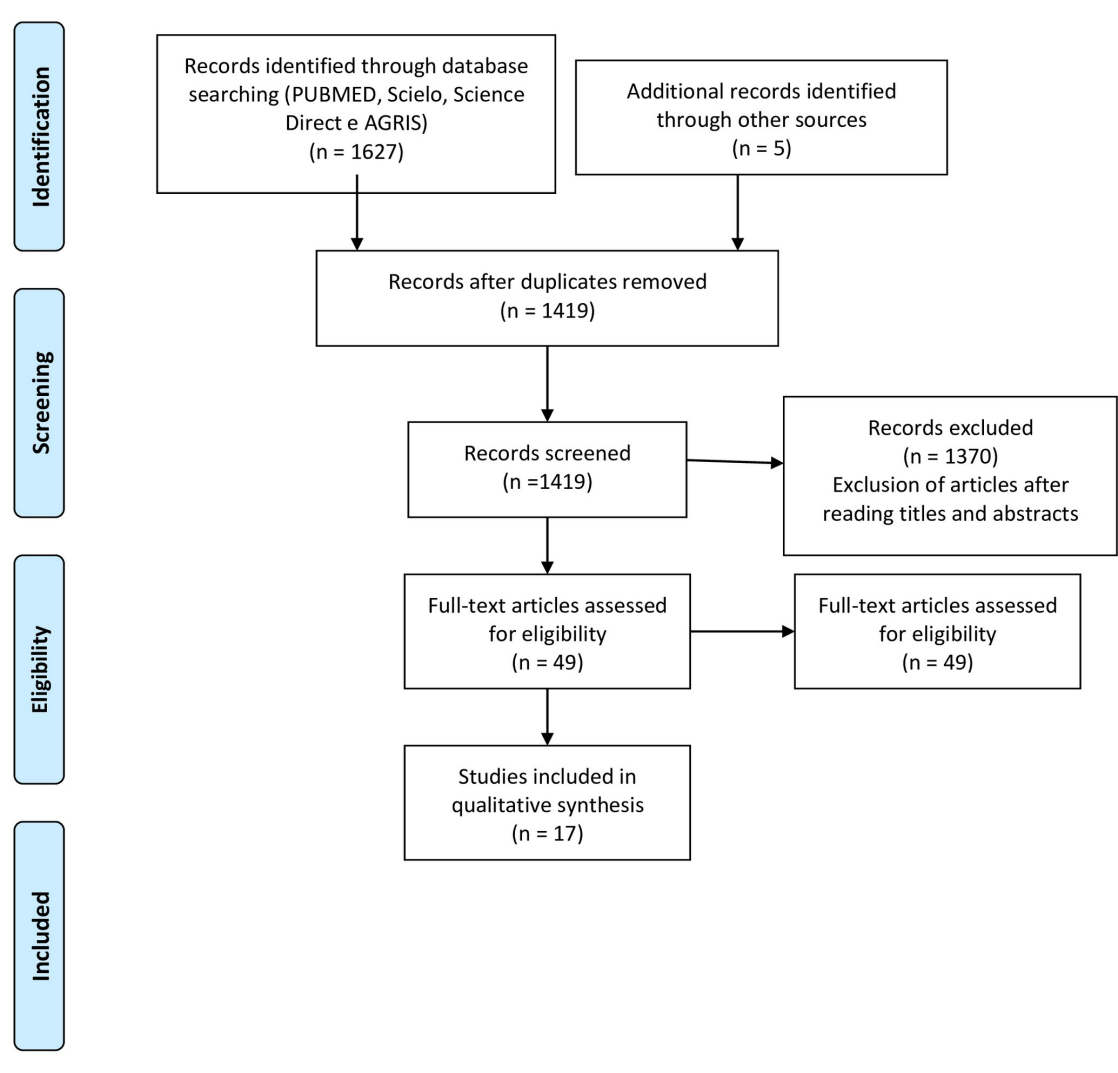
Prisma flow diagram. Preferred reporting items for systematic reviews and meta-analysis (PRISMA) flow diagram identifying the total number of articles initially surveyed, the number of articles included and excluded for this systematic review (Moher et al., 2009).

Details on the databases search and construction mechanisms are summarized in Fig 1 and are in line with the preferred reporting items for systematic reviews and meta-analyzes (Prisma) [18].

### Readability criteria

After the studies were selected, the articles underwent a thorough readability review according to a scoring scale of 0 (inadequate), 1 (inaccurate/partially adequate) and 2 (adequate), where only the most relevant items were scored, as shown. The scoring criteria, described below, were adapted from other published systematic reviews [19, 23, 24]:

- A—Sample n: the scoring system was established as follows: 0 (works with less than 4 animals/treatment), 1 (works with 4 to 19 animals/treatment) and 2 (works with more than 20 animals/treatment);
- B—Randomization: prospective studies conducted at random received 2 points, and those which were not randomized or whose experimental design was not clear received 1 point;
- C—Sample homogeneity: studies that used animals of the same breed, sex, size and age were considered homogeneous and received 2 points. Studies that did not use homogeneous samples or that did not mention one or more of the previous traits received 1 point;
- D—Intake levels: studies that mentioned the intakes of DM, OM, CP, EE and GE per animal received 2 points; studies that evaluated one to three of these variables received 1 point; and those which did not evaluate this parameter or in which results were not clear received 0 points;
- E—Digestibility coefficients: studies that measured the ADC of DM, OM, CP and EE per animal received 2 points; studies that evaluated one to three of these variables received 1 point; and those which did not evaluate these parameters or in which results were not clear received 0 points;
- F—Fecal characteristics: studies that evaluated fecal production and fecal dry matter received 1 point; studies that did not evaluate these variables received 0 points;
- G—Metabolizable energy: studies that complemented the digestibility results with the metabolizable energy values received 1 point, and those which did not measure this parameter received 0 points.

Additional data such as experimental period, fecal collection method, description of diet and ingredient processing, among others, were used only for descriptive purposes, without a scoring scale, and included in this study to contribute to the interpretation of results and synthesis of the discussion. After the articles were evaluated, 17 were selected for the present study.

## Results

The results were organized into groups according to the search in the indexing databases, totaling 1,627 articles (Fig 1). The largest number of journals (1,289) was concentrated in the PUBMED database, followed by Science Direct (196), AGRIS (131) and Scielo (11). The review of the articles began with the exclusion of duplicate articles, which resulted in 1,414 papers to be evaluated. Next, the titles were read and 967 works were excluded. Of the 447 articles remaining, 403 were excluded after the abstracts were read. The discarded articles corresponded to literature reviews, chapters of books, books and experiments in other biological areas. Finally, the remaining 44 papers were read in full and only 12 were in accordance with the established selection criteria. An additional search was performed through the references that were part of each of these works and another 5 new articles were found, resulting in 17 journals that were evaluated in this review.

Although most articles included in this review were published in English and in the last 19 years, there were no language or date restrictions during the search and selection of studies. Of the 17 articles included, 7 were local (Brazil) and 10 international (USA—6, Canada—1, Belgium—1, France—1 and United Kingdom—1), as shown in Table 3. An important finding was that no articles published before 2006 were found in the national literature, as the first studies started to be developed only from that date [13].

In addition to the evaluated variables that constitute the main objective of this systematic review, the inclusion levels and processing data of each ingredient were also analyzed. The quality of the selected articles was determined considering these criteria and measured using a score scale, as shown in Table 2. The maximum score assigned was 10 and the minimum was 4, out of 14 possible points. No study used less than 4 animals per treatment; half of the studies were randomized; and only 6 used homogeneous experimental groups, that is, animals of the same sex, size and/or age.

**Table 2:** Database of intake levels, nutrients digestibilty coefficients and fecal characteristic of dog protein ingredients according selected articles for systematic review.

#	Authos	Sample n	Age	Origin Country	Evaluated ingredients	Evaluated variables
1	Carciofi et al. (2009) [10]	18	Adults	Brazil	Soybean meal; Poultry offal meal;	**Intake**: DM, OM, CP, EE
						**ATTD**: DM, OM, CP, GE, ME, acid eter extract
					Micronized whole soybeans.	
						Fecal score, FDM, fecal wet;
						Palatability.
2	Carciofi et al.	24	Adults	Brazil	Soybean meal;	**ATTD**: DM, OM, CP, acid eter extract,
	(2006) [13]				Poultry offal meal;	
					Meat and bone meal;	FDM
					Corn gluten meal.	
3	Cavalari et al. (2006) [25]	4	Adults	Brazil	Whole soybean meal;	**ATTD**: DM, CP, GE; DE.
					Meat meal;	
					Extruded meat meal;	
					Poultry offal meal;	
					Extruded poultry meal	
					Extruded fish meal;	
					Extruded feather meal	
4	Clapper et al. (2001) [3]	6	Adults	USA	Soy protein concentrate;	**Intake**: DM, OM, CP, EE;
						**ATTD**: DM, OM, CP, EE, GE;
					Whole soy	
					protein;	Fecal production and fecal score.
					Soybean meal	
					Soy flour;	
					Poultry offal meal;	
5	Menniti et al. (2014) [26]	36	Adults	Canada	Soybean meal;	**Intake:** DM, CP;
						**ATTD:** DM, OM, CP, EE, ME, ED;
6	Maria et al. (2017) [24]	6	Adults and seniors	Brazil	Sugar cane fiber;	**Intake:** DM, ME;
						**ATTD**: DM OM, CP, EE, GE, ME.
					Beet pulp;	
						Fecal production, FDM, fecal score
					Soybean meal;	
					Poultry offal meal	
7	Bednar et al. (2000) [9]	4	Adults	USA	Soybean meal;	**Intake:** DM, OM, CP, EE,
						**ATTD**: DM, OM, CP, EE;
					Poultry meal;	
						Fecal production, FDM, fecal score.
					Poultry offal meal;	
					Meat and bone meal	
8	Félix et al. (2013) [22]	12	Adults and puppies	Brazil	Defatted soybean meal;	**Intake**: DM;
						**AATD**: DM, CP, EE, GE, ME;
						Fecal production, FDM, fecal wet, fecal score
					Soybeam meal;	
					Micronized soybeans;	
					Toasted soybeans	
9	Zanatta et al. (2013) [27]	8	Adults	Brazil	Soybean meal;	**ATTD**: DM, OM, CP, GE, ME, acid eter extract;
					Poultry offal meal	
						FDM, score fecal
10	Yamka et al. (2003) [12]	8	Adults	USA	Poultry offal meal	**Intake:** DM, CP;
						**ATTD**: DM, CP;
						Fecal production. FDM, fecal wet
11	Neirinck et al. (1991) [28]	4	Adults	Belgium	Soybean meal;	**ATTD**:DM, OM, CP, EE
					Lungs; offal; fresh meat	
12	Zuo et al. (1996) [29]	5	Adults	USA	Conventional Soybean meal;	**Intake**: DM, OM, CP, EE, GE;
						**ATTD:** DM, OM, CP, EE, GE
					Low oligosaccharide Soybean meal;	
					Poultry offal meal	
13	Kendall et al. (1982) [30]	6	Adults	United Kindom	Soybean meal;	**ATTD:** DM, OM, CP, GE;
						Fecal production.
					Textured soy protein;	
					Extracted soybean meal;	
					Full-fat soy flour; Micronized whole soybeans	
14	Murray et al. (1997) [31]	6	Adults	USA	Meat and bone meal;	**Intake**: DM, OM, CP, EE, GE
						**ATTD**: DM, OM, CP, EE, GE.
					Fresh beef meat;	
					Poultry offal meal;	
					Fresh poultry meat;	
					Deffated soybean	
					meal;	
					Dehydrated egg	
15	Yamka et al. (2006) [32]	6	Adults	USA	Soybean meal;	**Intake**: DM, GE
					Low oligosaccharide;	**ATTD**: DM, GE;
						Fecal production, fecal wet, FDM.
					Soybean meal;	
					Poultry offal meal	
16	Nery et al.	27	Adults	France	Corn gluten meal	**ATTD**: DM, CP, EE, GE
	(2010) [33]					Fecal production, fecal wet, fecal score, FDM.
					Poultry offal meal	
17	Tortola et al. (2013) [34]	24	Adults	Brazil	Poultry offal meal	**Intake**: DM, OM, CP. EE
						**ATTD**: DM, OM, CP, EE, GE, ME;
					Soybean meal	
					Soybean meal with enzyme addition.	Fecal production, fecal score, FDM

ATTD: apparent total tract digestibility; CP: crude protein; DM: dry matter; EE: ether extract; ED: digestible energy; FDM: fecal dry matter; GE: gross energy; ME: metabolizable energy.

The number of animals varied between studies (from 4 to 36 per treatment), totaling 204 animals in this review. Most studies evaluated digestibility coefficients in adult dogs and only 2 were comparative between ages [22, 24] (Table 2). From these 2, only the data compatible with the criteria established for this review were extracted.

Table 3 shows the inclusion levels of both soybean meal and poultry offal meal as well as other protein ingredients evaluated in the studies. Only two [28, 30] of the 17 studies did not provide this information. The processing of the ingredient and diet was only documented by two authors [3, 22].

**Table 3:** Inclusion levels of soybean meal, poultry offal meal and other protein sources evaluated in the studies and adults dogs diets processing information.

Authors	Selected ingredients inclusion levels (%)	Inclusion levels of other protein sources (%)	Ingredient processing	Diet processing
	***Soybean meal***			
Carciofi et al. (2009) [10]	29,5	12,0: meat and bone meal	NE	Evaluated
Carciofi et al. (2006) [13]	32,1	25,05: corn gluten meal	NE	NE
		29,22: meat and bone meal		
Clapper et al. (2001) [3]	44,0	17,1: chiken bone residue	NE	Evaluated
Menniti et al. (2014)	6,0;	20,8; 15,1 e 13,8: poultry offal meal	NE	NE
	11,5;			
		18,3: corn meal		
		6,5: fish meal		
	17,0			
Maria et al. (2017) [24]	30,0	11,2: poultry offal meal	NE	NE
Bednar et al. (2000) [9]	30,0	NE	NE	NE
Félix et al. (2013) [22]	46,7	59,8: maize	Evaluated	Evaluated
		26,14: poultry offal meal		
		5,71: corn gluten meal 60%CP		
Zanatta et al (2013) [27]	30,0	NE	NE	NE
Neirinck et al. (1991) [28]	NE	NE	NE	NE
Zuo et al. (1996) [29]	18,5;	NE	NE	NE
	37,1			
Kendall et al. (1982) [30]	9,1	NE	NE	NE
Yamka et al.(2006) [32]	30,9	NE	NE	NE
Tortola et al. (2010) [34]	30,0	NE	NE	NE
	***Poultry offal meal***			
Carciofi et al. (2009) [10]	22,8	12,0: meat and bone meal	NE	NE
Carciofi et al. (2006) [13]	23,7	NE	NE	NE
Cavalari et al. (2006) [25]	40,0	22,0 comemercial diet at proportion:	NE	NE
		60:40 corn gelatinized / poultry offal meal; and at		
		Proportion: 50:50 corn gelatinized / poultry offal meal		
Clapper et al. (2001) [3]	32,7	7,8 poultry offal meal	NE	Evaluated
Bednar et al. (2000) [9]	18,0	NE	NE	NE
Zanatta et al (2013) [27]	50,2	NE	NE	NE
Yamka et al. (2003) [12]	10,4;	NE	NE	NE
	17,8;			
	25,0;			
	32,5			
Zuo et al. (1996) [29]	33,3	NE	NE	NE
Murray et al. (1997) [31]	14,54	18,32: dehydrated egg	NE	NE
Yamka et al. (2006) [32]	22,4	NE	NE	NE
Nery et al. (2010) [33]	46,5	25,6 and 14,3: corn gluten meal	NE	NE
	74,4			
Tortola et al. (2013) [34]	28,9	NE	NE	NE

NE: none evaluated

The results described in Table 4 and Table 5 were obtained from a comparison between the effects of soybean meal and/or poultry offal meal and those of other protein ingredients evaluated in each scientific article.

**Table 4:** Inclusion levels and intake levels of dry matter (DM), crude protein (CP), ether extract (EE), gross energy (GE) of soybean meal and poultry offal.

Author/ year	Selected ingredients Inclusion levels (%)	Intake levels (g/kg/day)			Intake GE
					(kcal/day)
	*Soybean meal*	DM	CP	EE	
Carciofi et al. (2009) [10]	29,5	169,0*^↓^	42,0^NS^	21,0^NS^	NE
Clapper et al. (2001) [3]	44,0	299,0^NS^	95,0^NS^	76,0^NS^	1.641^NS^
Menniti et al. (2014) [26]	6,0	321,0*^↑^	93,3*^↑^	57,6*^↑^	NE
	11,5	318,0*^↑^	92,4*^↑^	57,5*^↑^	NE
	17,0	324,0*^↑^	97,9*^↑^	58,2*^↑^	NE
Maria et al. (2017) [24]	30,0	133,0^NS^	NE	NE	NE
Bednar et al. (2000) [9]	30,0	380,0^NS^	97,0^NS^	48^NS^	NE
Félix et al. (2013) [22]	46,7	246,0^NS^	NE	NE	NE
Zanatta et al. (2013) [27]	30,0	NE	NE	NE	NE
Neirinck et al. (1991) [28]	NE	NE	NE	NE	NE
Zuo et al. (1996) [29]	18,5	401,0^NS^	123,3^NS^	56,8*^↑^	2.005,0^NS^
	37,1	395,0^NS^	126,5^NS^	49,6*^↓^	1.947,0^NS^
Kendall et al. (1982) [30]	9,1	NE	NE	NE	NE
Yamka et al. (2006) [32]	30,9	331,0^NS^	NE	NE	4.649,0^NS^
Tortola et al. (2013) [34]	30,0	143,0^NS^	40,0^NS^	17,0*^↑^	NE
	***Poultry offal meal***				
Carciofi et al. (2009) [10]	22,8	189,0*^↑^	44,0*^NS^	22,0*^NS^	NE
Carciofi et al. (2006) [13]	23,7	NE	NE	NE	NE
Cavalari et al. (2006) [25]	40,0	NE	NE	NE	NE
Clapper et al. (2001) [3]	32,7	271,0^NS^	83,0^NS^	67,0^NS^	1.547,0^NS^
Bednar et al. (2000) [9]	18,0	338,0^NS^	83,0^NS^	48,0^NS^	NE
Zanatta et al. (2013) [27]	50,2	NE	NE	NE	NE
Yamka et al. (2003) [12]	10,4	278,8*^↓^	28,8*^↓^	NE	NE
	17,8	269,4*^↓^	40,6*^↓^	NE	NE
	25,0	294,5*^↓^	60,0*^↑^	NE	NE
	32,5	295,1*^↓^	73,8*^↑^	NE	NE
Zuo et al. (1996) [29]	33,3	400,9^NS^	121,6^NS^	59,0*^↑^	2.012,0^NS^
Murray et al. (1997) [31]	14,54	418,0^NS^	87,0^NS^	57,0^NS^	1.998,0^NS^
Yamka et al. (2006) [32]	22,4	304,0^NS^	NE	NE	4.819,0^NS^
Nery et al. (2010) [33]	46,5	NE	NE	NE	NE
	74,4	NE	NE	NE	NE
Tortola et al. (2013) [34]	28,9	135,0^NS^	37,0^NS^	17,0*^↓^	NE

CP: crude protein; DM: dry matter; EE: ether extract; ED: digestible energy; GE: gross energy; NE: none evaluated

NS: not significant

*↑: statistical differences (nutrient intake levels increased)

*: statistical differences (nutrient intake levels decreased).

**Table 5:** Inclusion effects of soybean meal and poultry offal meal under digestibility coefficients, metabolizable energy, fecal production and fecal dry matter.

Author / year	Selected ingredients Inclusion levels (%)	Apparent total tract digestibility (%)					Fecal production (g/d)	FDM (%)
	*Soybean meal*	DM	CP	EE	GE	ME		
Carciofi et al. (2009) [10]	29,5	84,0*^↑^	86,0^NS^	92,0^NS^	89,0^NS^	14,2*^↓^	318*^↓^	16,0^NS^
						(MJ/kg)		
Carciofi et al. (2006) [13]	32,1	81,1*^↓^	86,3^NS^	92,0^NS^	NE	NE	NE	30,1*^↓^
Menniti et al. (2014) [26]	6,0	80,2^NS^	80,9^NS^	91,0^NS^	NE	159%*^↑^	NE	32,4*^↓^
	11,5	80,9^NS^	82,1^NS^	91,8^NS^	NE	150%*^↑^	NE	30,8*^↓^
	17,0	81,4^NS^	83,1^NS^	92,0^NS^	NE	151%*^↑^	NE	30,2*^↓^
Maria et al. (2017) [24]	30,0	79,6^NS^	82,8*^↑^	90,0^NS^	85,5^NS^	4,03^NS^	114,2*^↑^	36,4^NS^
						(kcal/g)		
Bednar et al. (2000) [9]	30,0	78,3*^↓^	82,7*^↓^	88,4^NS^	NE	NE	226*^↑^	54.0*^↑^
Félix et al. (2013) [22]	46,7	75,8*^↓^	85,2*^↓^	NE	79,7*^↓^	17,0*^↓^	NE	31,1^NS^
						(MJ/kg MS)		
Zanatta et al. (2013) [27]	30,0	80,7^NS^	84,0*^↑^	90,7*^↓^	84,9*^↓^	4.198,7*^↓^	NE	29,1*^↓^
						(kcal/g/d)		
Neirinck et al. (1991) [28]	NE	73,8*^↓^	77,7*^↓^	89,4*^↓^	NE	NE	NE	NE
Zuo et al. (1996) [29]	18,5	77,7 ^NS^	80,3*^↑^	90,6 ^NS^	83,4 ^NS^	NE	NE	NE
	37,1	78,9 ^NS^	84,6*^↑^	90,8 ^NS^	84,1 ^NS^	NE	NE	NE
Kendall et al. (1928) [30]	9,1	75,0*^↓^	84,0^NS^	57,0*^↓^	78,0*^↓^	NE	NE	NE
Yamka et al. (2006) [32]	30,9	86,5*^↓^	NE	NE	NE	4.014,0*^↓^	142*^↑^	NE
						(kcal/g/d)		
Tortola et al. (2013) [34]	30,0	84,5^NS^	87,0^NS^	91,3^NS^	87,7^NS^	17,0^NS^	NE	30,7*^↓^
						(kJ/g MS)		
	***Poultry offal meal***							
Carciofi et al. (2009) [10]	22,8	83,0*^↓^	85,0^NS^	92,0^NS^	89,0^NS^	15,9*^↑^	454*^↑^	17,0*^↑^
						(MJ/kg)		
Carciofi et al. (2006) [13]	23,7	83,6*^↑^	84,4^NS^	91,7^NS^	NE	NE	NE	35,0*^↓^
Cavalari et al. (2006) [25]	40,0	88,1*^↓^	88,9*^↓^	NE	91,2*^↓^	NE	NE	NE
Clapper et al. (2001) [3]	32,7	81,9^NS^	76,9*^↓^	92,9*^↑^	84,9^NS^	NE	NE	30,0*^↓^
Bednar et al.(2000) [9]	18,0	84,5*^↑^	87,5*^↑^	91,9^NS^	NE	NE	117,0*^↓^	39,0*^↓^
Zanatta et al. (2013) [27]	50,2	81,2^NS^	82,7*^↓^	94,8*^↑^	87,9*^↑^	4.464,8*^↑^	NE	41,9*^↑^
						(kcal/g/d)		
Yamka et al. (2003) [12]	10,4	91,2^NS^	81,0*^↓^	NE	NE	NE	NE	24,8*^↑^
	17,8	90,8^NS^	84,5*^↓^	NE	NE	NE	NE	25,2*^↑^
	25,0	90,3^NS^	86,3*^↓^	NE	NE	NE	NE	28,6*^↑^
	32,5	89,4^NS^	86,6*^↑^	NE	NE	NE	NE	31,6*^↑^
Zuo et al. (1996) [29]	33,3	77,0 ^NS^	77,2*^↓^	90,6 ^NS^	83,1 ^NS^	NE	NE	NE
,Murray et al. (1997) [31]	14,54	85,1^NS^	89,5^NS^	93,7^NS^	92,1^NS^	NE	NE	NE
Yamka et al. (2006) [32]	22,4	91,3*^↑^	NE	NE	NE	4.254,0*^↑^	64,8*^↓^	NE
						(kcal/g/d)		
Nery et al. (2010) [33]	46,5	84,4*^↑^	81,3*^↓^	96,3^NS^	88,3*^↑^	NE	658,0^NS^	NE
	74,4	82,4*^↓^	83,1*^↑^	95,5^NS^	85,9*^↓^	NE	665,0^NS^	NE
Tortola et al. (2013) [34]	28,9	85,6^NS^	85,9^NS^	91,7^NS^	88,1^NS^	17,4^NS^	NE	37,0*^↑^
						(kJ/g MS)		

CP: crude protein; DM: dry matter; EE: ether extract; ED: digestible energy; FDM: fecal dry matter; GE: gross energy; ME: metabolizable energy; NE: none evaluated

NS: not significant

*↑: statistical differences (nutrient apparent total tract digestibility increased)

*: statistical differences (nutrient apparent total tract digestibility decreased).

In most studies that evaluated soybean meal, there were no statistical differences for the intakes of DM (g/kg/day) [3, 9, 22, 24, 29, 32, 34], CP (g/kg/day) [3, 9, 10, 29, 34], EE (g/kg/day) [3, 9, 10], or GE (kcal/day) [3, 29, 32]. The same was observed for the intakes of DM [3, 9, 29, 31, 32, 34], CP [3, 9, 10, 29, 31, 34], EE [3, 9, 10, 31] and GE [3, 29, 32] from poultry offal meal. In the studies in which differences were detected between treatments, both for soybean meal and poultry offal meal, there was great variability in the results and a close relationship was observed between the intake levels and the ingredient inclusion levels, i.e., higher inclusion levels resulted in higher intakes of DM, CP, EE and GE and vice-versa (Table 4).

As regards the digestibility coefficient (Table 5), in most studies, the inclusion of soybean meal reduced the ADC of DM [9, 13, 22, 28, 30, 32], EE [3, 27, 28, 30] and GE [22, 27, 30] and increased the ADC of CP [3, 24, 27, 29]. Of the 13 articles cited, 7 [10, 22, 24, 26, 27, 32, 34] presented the metabolizable energy (ME) values of the diet and, within these studies, soybean meal inclusion reduced ME.

In contrast to what was observed for soybean meal, most studies that investigated poultry offal meal did not describe statistical differences in the digestibility results [3, 12, 27, 29, 31, 34]. Only one study [33], in which different inclusion levels were tested, showed opposite results, i.e., when the level of inclusion of the ingredient was increased, the ADC of DM decreased, whereas when its inclusion level was reduced, the ADC of DM increased. The ADC of CP was the variable that most varied in the results: 4 articles reported no statistical differences [10, 13, 31, 34]; another 4 [3, 25, 27, 29] showed reduced digestibility; and only one [9] described an increase in the coefficient. Results were divergent in only two articles [12, 33], in which the authors compared increasing levels of inclusion and observed that higher levels resulted in increased digestibility of the ingredient, whereas lower levels induced a reduction in the ADC of CP. For the ADC of EE, no significant differences were described in most studies [9, 10, 13, 29, 31, 33, 34]. As with soybean meal, the ME value was not specified in most (9) of the studies cited, and the results found in the journals that evaluated this parameter [9, 10, 32, 33] were divergent, making it difficult to interpret the data.

For soybean meal, fecal production was not evaluated in most (9) of the articles found. In contrast, fecal dry matter (FDM) was analyzed in 9 of the articles, and the inclusion of the ingredient reduced FDM in 4 of these studies [13, 26, 27, 34]. For poultry offal meal, the same was observed in terms of fecal production, with the majority of articles (8) not analyzing this parameter. Fecal dry matter was evaluated in 7 articles, and the inclusion of the ingredient increased FDM in 4 of these studies [10, 12, 27, 34].

It is worth stressing that all the studies included in this review evaluated the composition and digestibility of the experimental diet only, not the ingredient, specifically. In addition, the ME values described in Table 5 are expressed in different units, since this variable was measured and described according to the methodology of each author.

## Discussion

Systematic reviews use specific methodologies to undertake a complete literature search, allowing a broad and clear visualization of the results of a given subject over several years. In this way, it offers impartial suggestions on the best methodological protocols to be employed or on the implementation of new lines of research, directing the researcher to more objective conclusions.

Commercial dog foods are made up of various protein components, both animal- and plant-derived. However, when we conducted a pre-review to evaluate the state of the art in the subject, results led to a new search for two specific ingredients (soybean meal and poultry offal meal). These were chosen because they are the main protein sources used in the formulation of dog foods and also because the number of studies with other protein ingredients (meat-and-bone meal and maize by-products) was limited, which would make it difficult to draw any conclusions about their effects.

The criteria for checking the quality of the selected articles were the presence or absence of randomization, number of animals per treatment, homogeneity of the studies and complete availability of the data for each variable measured (ADC, intake, fecal production and FDM). As an essential part of the results, no study was identified as a blind experiment so as to reduce any type of bias during the experimental protocols, providing greater credibility to the results. In addition, sample size is another important factor to be considered; most studies used less than 10 animals per treatment.

In digestibility trials, some fundamental factors inherent to the animal must be considered, e.g., species, age, size and physiological condition. Therefore, for this review, it was appropriate to evaluate studies solely with medium-sized and healthy adult animals (up to 6 years old).

Dogs are considered *puppies* until 1 year old, adult between 1 and 6 years old and senior from 7 years of age [21], and each phase of life has a specific nutritional requirement. For instance, puppy dogs have a 50% higher calorie requirement in their diet than adult dogs [21, 22]. Senior dogs, on the other hand, do not have specific nutritional needs, which are equivalent to that of an adult animal [35]. This aspect is even more important in nutrition studies, since digestibility varies according to the animal’s energy requirement [22].

As previously mentioned, digestibility varies with animal age, physiological condition and size. Large dogs are prone to produce lower-quality stools (poorly formed, moist and fetid) and a larger fecal volume [36, 37]. This can be explained by the anatomical and physiological differences between sizes. One of these differences can be seen in the larger area of relative absorption, which, associated with the volume of the intestinal tract and higher rate of colonic fermentation, results in reduced absorption of electrolytes and water, directly impacting digestibility and fecal characteristics [38]. This is one of the reasons why dogs of other sizes were not included in this review.

Considering the evaluated protein sources, factors such as the origin and processing of the ingredients are crucial to interpret the different results found for the digestibility of the food. In this study, seven articles were national and the others were published in several countries, which is important, since the nutritional composition of the raw material varies according to the country of origin, meaning there is no standardization [38, 39]. Moreover, each supplier employs different processing methods and quality standards for each ingredient. These methodological and quality differences result in products with an even more variable composition, which will directly interfere with the digestibility and bioavailability of nutrients [12]. Nevertheless, soybean meal has a more uniform composition and its processing conditions vary less between suppliers when compared with poultry meal [34, 40].

Based on most of the results described in the selected studies, there were no statistical differences in the intake levels of the analyzed variables between soybean meal and offal meal. Feed intake is influenced by several factors, among which are the physicochemical characteristics (flavor, texture, aroma and taste) and energy density of the diet. In other words, foods with a higher energy concentration (whether it comes from protein, lipids or carbohydrates) are consumed in smaller quantities than lower-energy foods. This is demonstrated in some studies that examined different inclusion levels [12, 41].

In addition to feed intake, other important complementary data to be considered are fecal production and FDM. Despite the large variability of results, overall, soybean meal was found to reduce FDM, whereas poultry offal meal tends to increase this coefficient. Other factors can influence the FDM increase, some of the factors are diet dry matter intake, the digestibility of nutrients present in the raw material, sources of the ingredients and process type. Therefore, food intake increase may not always lead to higher levels of FDM production [12, 42, 43]. Additionally, fecal production generally reflects the concentration of indigestible diet components [27, 44]. For instance, soybean meal contains oligosaccharides (raffinose, stachyose, b-mannanase), phytates, hemicellulose and non-structural carbohydrates that are excessively fermented by the intestinal microbiota [3, 12]. Fermentation results in the production of short-chain fatty acids, which increase intraluminal osmotic pressure and contribute to reducing fecal dry matter and increasing its moisture [9, 12]. In the case of diets based on poultry offal meal, results are even more inconsistent due to wide variations in the composition of the product. Meals with high ash and low protein contents lead to greater mineral losses (calcium, phosphorus, magnesium) through the feces, consequently increasing the FDM content [45, 46].

Finally, the inclusion of the two protein sources in the foods resulted in different effects on digestibility. As demonstrated in most studies, soybean meal reduces the digestibility coefficients of DM, OM, EE, GE and ME. The presence of antinutritional factors (protease inhibitors) in soybean can depress the bioavailability of nutrients [47]. In contrast, the ADC of CP increases with the inclusion of the raw material in the diet. This may be related, in part, to the proper processing of the ingredient and the food, since the thermal process inactivates the anti-nutritional factors present in the meal [26]. When properly processed, soybean meal constitutes an excellent source of protein [3, 29], although must be associated with other protein sources to ensure the adequate intake of all amino acids essential to the species [10, 13, 32], as described in all the studies cited.

With poultry offal meal, the effects were opposite for the ADC of DM, OM and ME, which increased with the inclusion of the ingredient. No significant differences were seen for the ADC of EE and GE, whereas the ADC of CP decreased. The remarkable variation of results reflects the lack of uniformity in the composition of the ingredient. The studies that showed better digestibility coefficients likely used a meal with less variation in its composition and with higher proportions of more digestible components (offal, muscle tissue, adipose tissue) [12, 13]. Conversely, those which reported the worst digestibility coefficients for the protein indicate that the meal used possibly had a higher concentration of minerals and a lower proportion of protein [25, 31].

## Conclusion

The results of this review demonstrate the superior effect of poultry offal meal over soybean meal on the digestibility of dietary nutrients in adult dogs. We recommend evaluating and comparing these ingredients at different inclusion levels, under standardized study conditions, so that less variable results are obtained and the existence of specific effects for each treatment confirmed, since the evaluated methodologies were highly heterogeneous. Therefore, trials should include the minimum necessary information, such as an assessment of the impacts of using different raw-material inclusion levels, the same feeding period, data on the collection of biological material and information on the processing of the ingredient and of the experimental diet.

## Supporting information

S1 File Prisma cheklist. (ZIP)

## References

[1] Association of American Feed Control Officials. Dogs and cats nutriente profiles. In: AAFCO Official Publication. AFFCO, Washington, D.C, USA, 2010; 169–183

[2] Case LP, Carey DP, Hirakawa DA, et al. Canine and feline nutrition. A resource for companion animal professionals. 2 st ed. St. Louis: Moshy, 2000. pp. 592

[3] Clapper GM, Grieshop CM, Merchen NR, et al. Ileal and total tract nutriente digestibilities and fecal characteristics of dogs as affect by soybean protein inclusion in dry, extrused diets. J. Anim. Sci. 2001; 79: 1523–1532. 10.2527/2001.7961523x 11424690

[4] Hendrix DL. Rapid extraction and analysis of nonstructural carbohydrates in plant tissues. Crop. Sci. 1993; 25:984–989. 10.2135/cropsci1993.0011183X003300060037x

[5] Gajda M, Flickinger CM, Grieshop L, et al. Corn hybrid affects in vitro and in vivo measures of nutrient digestibility in dogs. J. Anim. Sci. 2005; 83: 160–171. 10.2527/2005.831160x 15583056

[6] De Oliveira LD, Picinato MAC, Kawauchi IM, Sakomura NK, Carciofi, AC. Digestibility for dogs and cats of meat and bone meal processed at two different temperature and pressures levels. J. Anim. Physiol. Anim. Nutr. 2012; 96:1136–1146. 10.1111/j.1439-0396.2011.01232.x 21954906

[7] Faber TA, Bechtel PJ, Hernot DC, et al. Protein digestibility evaluations of meat and fish substrates using laboratory, avian, and ileally cannulated dog assays. J. Anim. Sci. 2010; 88:1421–1432. 10.2527/jas.2009-2140 20023140

[8] Brown WY, Vanselow BA, Redman AJ, Pluske JR. An experimental meat-free diet maintained haematological characteristics in sprint-racing sled dogs. Br. J. Nutr. 2009; 102:1318–1323. 10.1017/S0007114509389254 19480731

[9] Bednar GE, Murray SM, Patil AR, et al. Selected animal and plant protein sources affect nutriente digestibiity and fecal characteristics of ileally cannulated dogs. Arch. Anim. Nutr. 2000; 53:127–140. 10.1080/17450390009381942 10849867

[10] Carciofi AC, De Oliveira LD, Valério AG, et al. Comparison of micronized whole soybeans to common protein sources in dry dog and cat diets. Anim. Feed Sci. Tech. 2009; 151:251–260. 10.1016/j.anifeedsci.2009.01.00210.1016/j.anifeedsci.2009.01.002

[11] Jhonson ML, Parsons CM. Effects of raw material source, ash contente, and assay length on protein effeciency ratio and net protein ratio values for animal protein. Poult. Sci. 1997; 76: 1722–1727. 10.1093/ps/76.12.1722 9438288

[12] Yamka RM, Jamikorn U, True AD, Harmon DL. Evaluation of low-ash poultry meal as a protein source in canine foods. J. Anim. Sci. 2003; 81:2279–2284. 10.2527/2003.8192279x 12968703

[13] Carciofi AC, Pontieri R, Ferreira CF, Prada F. Avaliação de dietas com diferentes fontes proteicas para cães adultos. R. Bras. Zootec. 2006; 35:754–760. ISSN: 1806-9290

[14] Purushotham B, Radhakrishna PM, Sherigara BS. Effects of steam conditioning and extrusion temperature on some anti-nutriticional factors of soyabean (glycine max) for pet food applications. Am. J. Anim. Sci. 2007; 2:1–5. 10.3844/ajavsp.2007.1.5

[15] Dust JM, Grieshop CM, Parsons CM, et al. Chemical composition, protein quality, palatability and digestibility of alternative protein sources for dogs. J. Anim. Sci. 2005; 83:2414–2422. 10.2527/2005.83102414x 16160054

[16] Beloshapka NA, Godoy MRC, Detweiler KB, et al. Apparent total tract macronutrient digestibility, fecal characteristics, and fecal fermentative en-product concentrations of healthy adult dogs fed bioprocessed soy protein. J. Anim. Sci. 2016; 94:3826–3834. 10.2527/jas.2016-0449 27898907

[17] Hagely KB, Palmquist D, Bilyeu KD. Classification of distinct seed carbohydrate profiles in soybean. J. Agric. Food Chem. 2013; 61:1105–1111. 10.1021/jf303985q 23317449

[18] Libearti A, Altman DG, Tetzlaff J, et al. The PRISMA statemente for reporting systematic reviwes and meta-analyses of studies that evaluate health care interventions: explanation and elaboration. Plos Med. 2009; 6:1–28. 10.1371/journal.pmed.1000100 PMC2707010 19621070

[19] Palencia JY, Saraiva A, Abreu MLT, et al. Effectiveness of citruline and N-carbamoyl gluatamate as arginine precursors on reproductive performance in mammals: A systematica review. Plos One. 2018; 13:1–20. 10.1371/journal.pone.0209569 PMC6301651 30571792

[20] Bermingham EN, Thomas DG, Cave NJ, et al. Energy requirements of adult dogs: a meta-analysis. Plon One. 2014; 9: 1–23. 10.1371/journal.pone.0109681 25313818 PMC4196927

[21] NRC (Nacional Research Council). Nutrient requirements of dogs and cats. Washington, D.C, USA: In National Academy. 2006; pp 426.

[22] Félix AP, Zanatta CP, Brito, CBM, et al. Digestibility and metabolizable energy of raw manufactured with different processing treatments and fed to adult dogs and puppies. J. Anim. Sci. 2013; 91:2794–2801. 10.2527/jas.2011-4662 23572259

[23] Ferreira L, Lisenko K, Barros B, et al. Influence of médium-chain triglycerides on consumption and weight gain in rats: a systematica review. J. Anim. Physiol. Anim. Nutr 2012; 94:1–8. 10.1111/jpn.12030 23298149

[24] Pereira UP, Oliveira DGS, Mesquita LR, et al. Efficacy of Staphylococcus aureus vacines for bovine mastites: A systematica review. Vet Microbio. 2011; 148:117–124. 10.1016/j.vetmic.2010.10.003 21115309

[25] Murray SM, Patil AR, Fahey GC Jr, et al. Raw and rendered animal by-products as ingredients in dog diets. J. Ani. Sci. 1997; 75:2497–2505. 9303468

[26] Bellaver C. Limitações e vantagens do uso de farinhas de origem animal na alimentação de suínos e de aves. In: 2° Simpósio Brasileiro Alltech da Indústria de Alimentação Animal. Curitiba, Paraná, 2005. http://www.cnpsa.embrapa.br/sgc/sgc_publicacoes/publicacao_u5u82m5u.pdf

[27] Menniti MF, Davenport GM, Shoveller Ak, et al. Effect of graded inclusion of dietary soybean meal on nutrient digestestibility, health, and metabolic índices of adult dogs. J. Anim. Sci. 2014; 92:2094–2104. 10.2527/jas.2013-7226 24668960

[28] Maria APJ, Ayane L, Putarov TC, et al. The effect of age and carbohydrate and protein sources on digestibility, fecal microbiota, fermentation products, fecal IgA, and immunological blood parameters in dogs. J. Ani, Sci. 2017; 95:2452–2466. 10.2527/jas2016.1302 28727033

[29] Yamka RM, Harmon DL, Schoenherr WD. In vivo measurement of flatulence and nutriente digestibility in dogs fed poultry by-product meal, conventional soybean meal, and low-oligosaccharide low-phytate soybean meal. Am. J. Vet. Res. 2006; 67:88–94. 10.2460/ajvr.67.1.88 16426217

[30] Neirinck K, Istasse L, Gabriel A, et al. Amino acid composition and digestibility of four protein sources for dogs. J. Nutr. 1991; 121:64–65. 10.1093/jn/121.suppl_11.S64 1941241

[31] Zuo Y, Fahey GC Jr, Merchen NR, Bajjalieh NL. Digestion responses to low oligosaccharide soybean meal by ilealli-cannulated dogs. J. Anim. Sci. 1996; 74:441–2449. 10.2527/1996.74102441x 8904713

[32] Zanatta CP, Gabeloni LR, Félix AP, et al. Metodologias para determinação da digestibilidade de dietas contendo fontes proteicas vegetal ou animal em cães. Cienc. Rural. 2013; 43:696–701. ISSN 0103-8478

[33] Nery J, Biourge V, Tournier C, et al. Influece of dietary protein contente and source on fecal quality electrolyte concentrations, and osmolarity, and digestibility in dogs differing in body size. J. Anim. Sci. 2010; 88:156–196. 10.2527/jas.2008-1666 19854997

[34] Kendall P, Holme DW. Studies on the digestibility of soya bean products, cereals, cereal and plant by-products in diets of dogs. J. Sci. Food. Agric. 1982; 33:813–822. 10.1002/jsfa.2740330902

[35] Tortola L, Souza NG, Zaine, L, et al. Enzyme effects on extruded diets for dogs with soybean meal as a substitute for poultry by-product meal. J. Anim. Physiol. Anim. Nutr. 2013; 97:39–50. 10.1111/jpn.12009 23639016

[36] Weber M, Martin L, Biourge V, et al. Influence of age and body size on the digestibility of a dry expanded diet in dogs. J. Anim. Physiol. Anim. Nutr. 2003; 87:21–31. 10.1046/j.1439-0396.2003.00410.x 14511146

[37] Meyer H, Zentek J, Habernoll H, Maskell I. Digestibility and compatibility of mixed diets faecal consistency in diferente breeds of dog. Zentralbl. Veterinarmed A. 1999; 46:155–165. 10.1046/j.1439-0442.1999.00201.x 10337231

[38] Sunvold GD, Fahey GC, Merchen NR, et al. Dietary fiber for dogs: IV In vitro fermentation of selected fiber sources by dog fecal inoculum and in vivo digeetsion and metabolismo of fiber-supplemente diets. J. Anim. Sci. 1995; 73:1099–1109. 10.2527/1995.7341099x 7628954

[39] Parsons CM, Zhang Y, Araba M. Nutritional evaluation of soybean meals in oligossacharide content. Poul. Sci. 2000; 79:1127–1131. 10.1093/ps/79.8.1127 10947181

[40] Stroucken et al. Extruding vc pelleting of a feed mixture lowers apparent nitrogen digestibility in dogs. J. Sci. Food Agric. 1996; 71:520–522. 10.1002/(SICI)1097-0010(199608)71:4<520::AID-JSFA612>3.0.CO;2-X

[41] Mendes WS, et al. Composição química e valor nutritivo da soja crua e submetida a diferentes processamentos térmicos para suínos em crescimento. Arq. Bras. Med. Vet. Zoo. 2004; 56:207–213. 10.1590/S0102-09352004000200011

[42] Taylor EJ, Adams C, Neville R. Some nutritional aspects of ageing in dogs and cats. Proc. Nutr. Soc. 1995; 54:645–656. 10.1079/pns19950064 8643702

[43] Camargo GP. Patil A, Cupp CJ, Malnoe A. Method and dietary composition for improving fat digestibility. Patent application publication, USA. 2005.

[44] Félix AP, Brito CBM, Ferrarini H, et al. Características físico-químicas de derivados proteicos da soja em dietas extrusadas para cães. Cienc. Rural. 2010; 40:256–2573. 10.1590/S0103-84782010001200021

[45] Laflamme DP. Nutriotional care for aging cats and dogs. Vet. Clin North Am Small Anim. Pract. 2012; 42:769–791. 10.1016/j.cvsm.2012.04.002 22720813

[46] Masuoka H, et al. Transition of the intestinal microbiota of dogs with age. Biosci. Microbiota Food Health. 2016; 36:27–31. 10.12938/bmfh.BMFH-2016-021 PMC5301054 28243548

[47] Ar Jadad, Moore RA, Carroll D, et al. Assenting the quality of reports of randomized clinical trials: is blinding necessary? Control Clin. Trials. 1996; 17:1–12. 10.1016/0197-2456(95)00134-4 8721797

