# Supplementary figures and images for: Soybean meal and poultry offal meal effects on digestibility of adult dogs diets: Systematic review
Source: PLoS One. 2021 May 27;16(5):e0249321. doi: 10.1371/journal.pone.0249321 (PMC8158863; doi:10.1371/journal.pone.0249321)

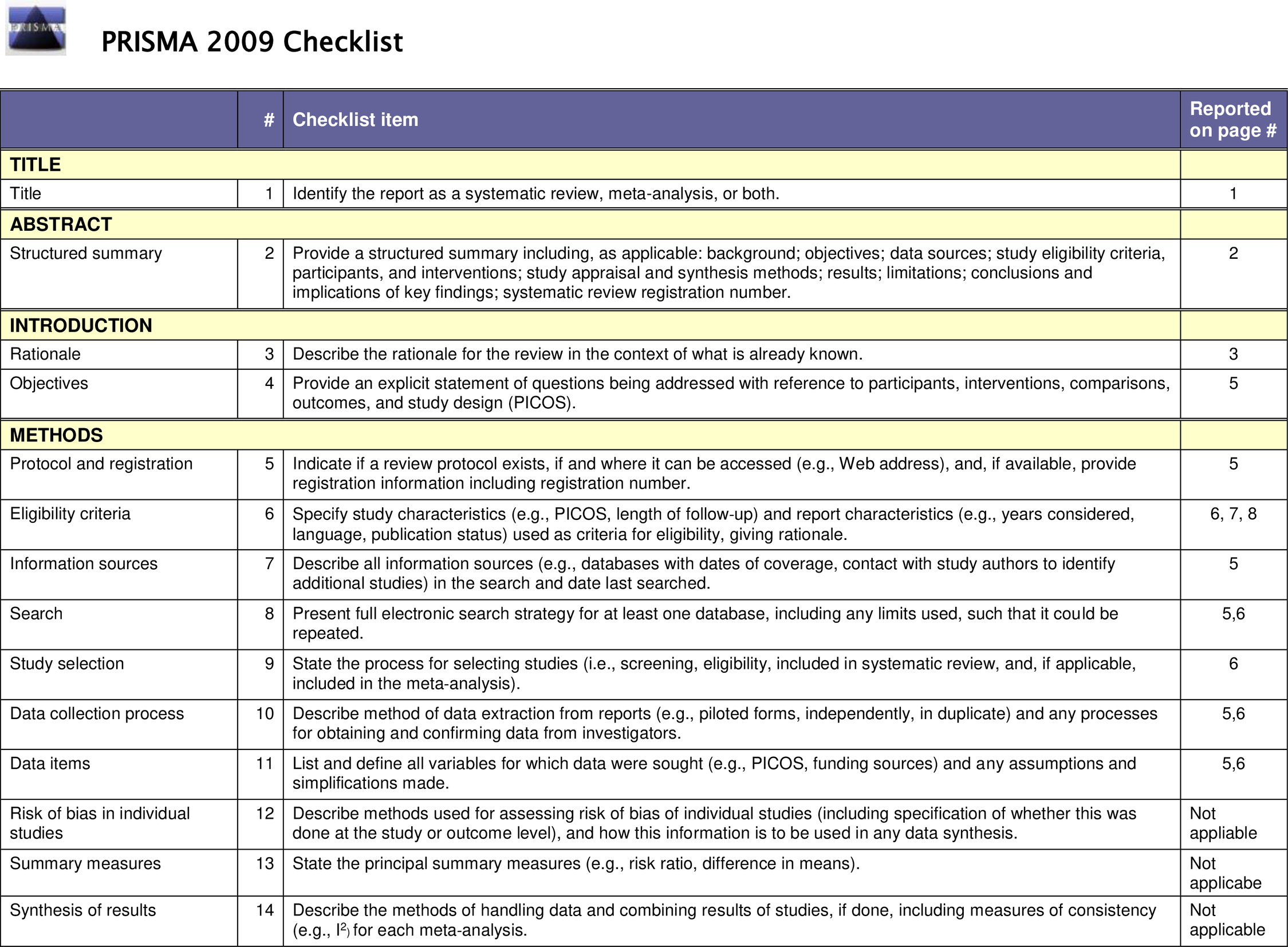

Supplement: S1 File — (ZIP) [file pone.0249321.s001.zip › S1_Checklist (A).tif]

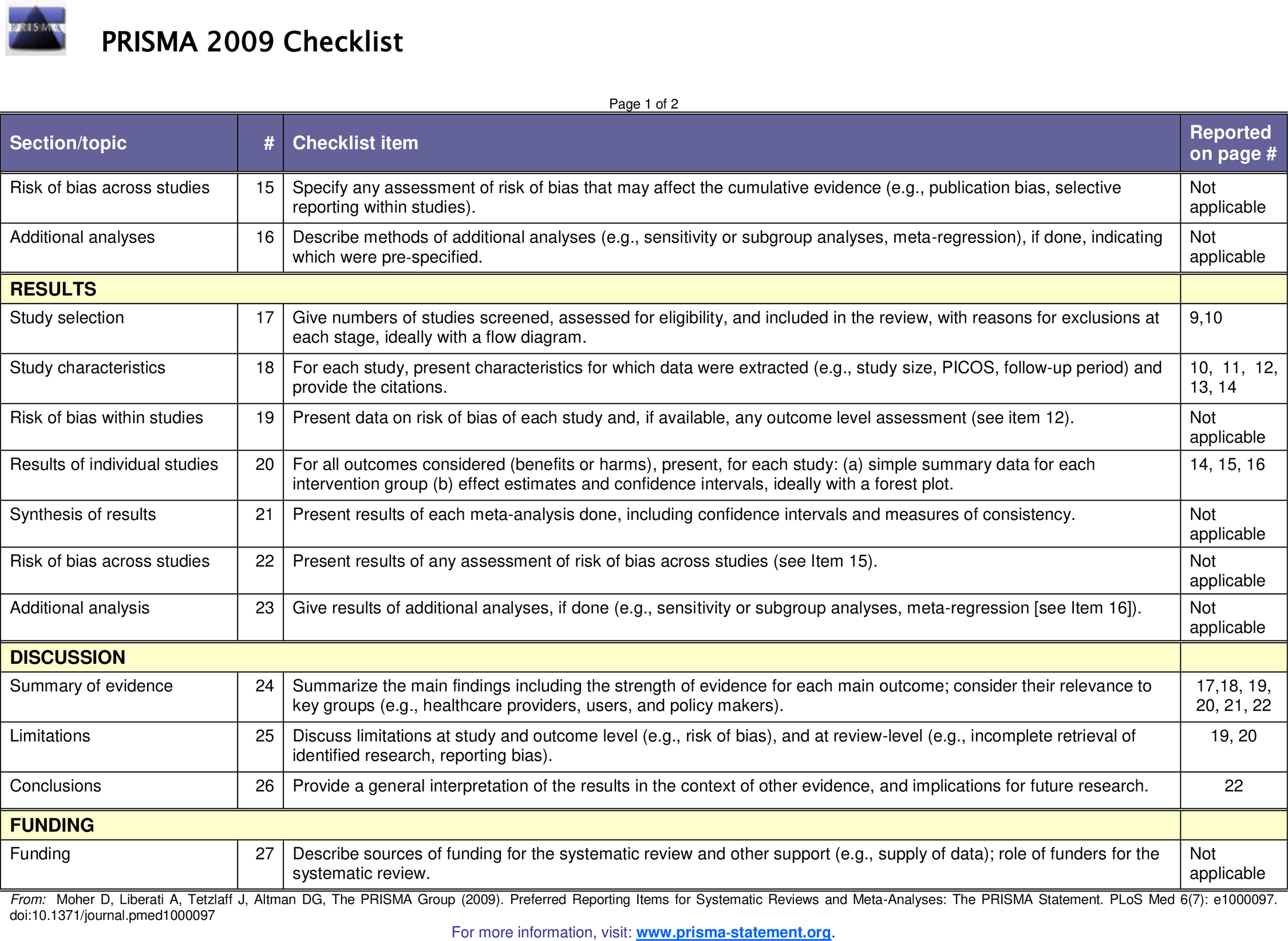

Supplement: S1 File — (ZIP) [file pone.0249321.s001.zip › S1_Checklist (B).tif]

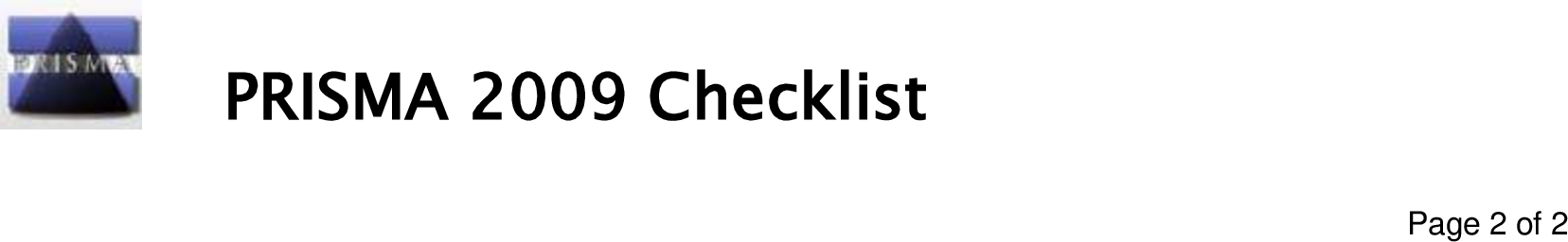

Supplement: S1 File — (ZIP) [file pone.0249321.s001.zip › S1_Checklist (C).tif]
